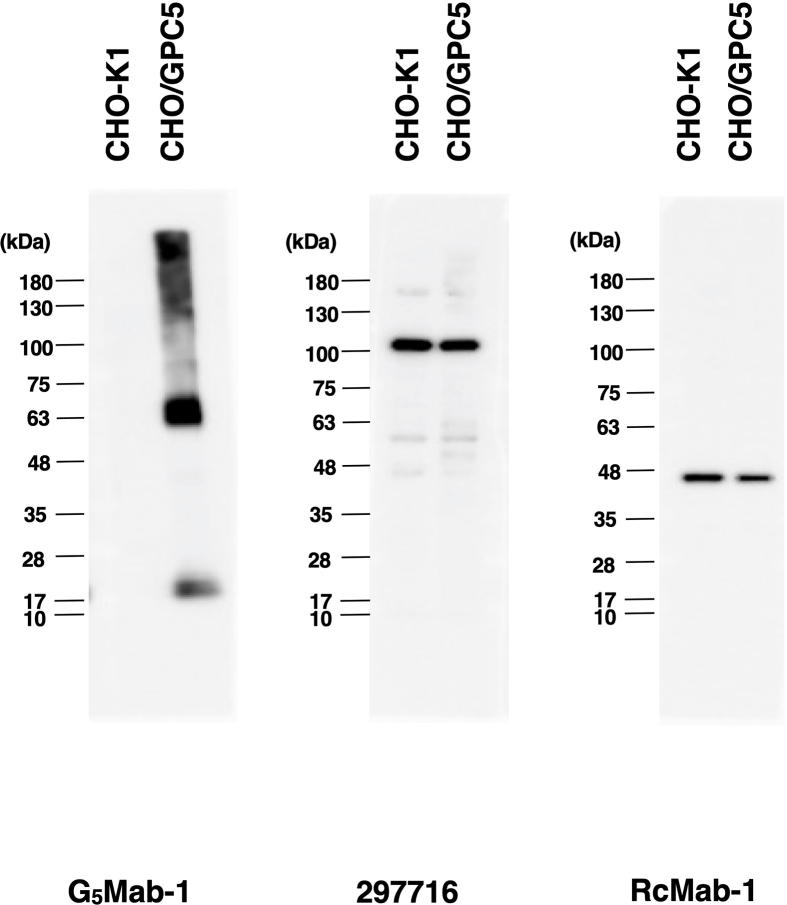# Development of a novel anti-human glypican 5 monoclonal antibody (G_5_Mab-1) for multiple applications

**DOI:** 10.1016/j.bbrep.2025.102140

**Published:** 2025-07-09

**Authors:** Yu Kaneko, Tomohiro Tanaka, Shiori Fujisawa, Guanjie Li, Hiroyuki Satofuka, Mika K. Kaneko, Hiroyuki Suzuki, Yukinari Kato

**Affiliations:** Department of Antibody Drug Development, Tohoku University Graduate School of Medicine, 2-1, Seiryo-machi, Aoba-ku, Sendai, Miyagi, 980-8575, Japan

**Keywords:** Glypican 5, Cell-Based Immunization and Screening method, Monoclonal antibody, Flow cytometry

## Abstract

Glypican 5 (GPC5) is a member of heparan sulfate proteoglycans and is anchored to the plasma membrane via glycosylphosphatidylinositol. GPC5 plays an essential role in kidney, limb, and brain development. Furthermore, GPC5 is expressed in some cancers, but whether it functions as a cancer-promoting or -suppressing factor remains unclear. Therefore, the development of versatile and specific anti-GPC5 monoclonal antibodies (mAbs) is desired to clarify the biological and pathological functions of GPC5. In this study, we successfully established an anti-human GPC5 mAb (clone G_5_Mab-1) using the Cell-Based Immunization and Screening method. G_5_Mab-1 is capable of using flow cytometric analysis. G_5_Mab-1 is specifically bound only to GPC5, not to the other GPC family members. The dissociation constant value of G_5_Mab-1 for GPC5-overexpressed Chinese hamster ovary K-1 (CHO/GPC5) cells was determined as 9.9 × 10^−9^ M. Furthermore, G_5_Mab-1 detected GPC5 in Western blot and immunohistochemistry using CHO/GPC5 cells. Therefore, the G_5_Mab-1 is highly versatile for basic research and is expected to contribute to clinical applications, such as antibody-based therapy and diagnosis of cancer.

## Highlights

• A novel anti-human glypican 5 (GPC5) monoclonal antibody (G_5_Mab-1) was developed by the Cell-Based Immunization and Screening method.
• G_5_Mab-1 is useful for the detection of human glypican 5 by flow cytometry, western blotting, and immunohistochemistry.
• G_5_Mab-1 did not show cross-reactivity to other glypicans.
• G_5_Mab-1 is expected for clinical applications including tumor diagnosis and therapy.

## 1 Introduction

The glypican (GPC) family is a member of glycophosphatidylinositol (GPI)-anchored heparan sulfate proteoglycans, consisting of six molecules, GPC1 to GPC6 [1,2]. GPCs comprise a core protein of approximately 60–70 kDa with a three-dimensional structure formed by multiple disulfide bonds to which heparan sulfate chains are attached. The core of GPCs is bound to the plasma membrane by GPI [3]. The amino acid sequences of the six vertebrate GPCs are 17%–63% identical [2,4]. GPCs have been thought to act as co-receptors and mediate several signaling pathways, including Wnt, hedgehog, and fibroblast growth factor (FGF) [[5], [6], [7], [8]]. GPCs play pivotal roles in cell growth, development, and some disorders such as cancer and multiple sclerosis [1,[9], [10], [11], [12], [13]].

GPC5 was characterized in 1997 as the gene located in chromosome 13q32 and expressed in the human adult brain [14]. In embryonic development, GPC5 expression is observed in central nervous system, kidney, and limb [9]. GPC5 is functional in normal tissues from an early stage. GPC5 gene amplification and protein overexpression are observed in malignant lymphoma, non-small cell lung cancer (NSCLC), breast cancer, and rhabdomyosarcoma [[15], [16], [17], [18]]. High GPC5 expression contributes to poor prognosis of NSCLC [16]. GPC5-mediated activation of several pathways, such as Wnt, hedgehog, and FGF, might be involved in tumor development and progression [2,6,19,20].

In contrast, GPC5 has also been considered as a tumor suppressor. The lower expression of GPC5 is observed in various tumor types, including hepatocellular carcinoma, NSCLC, prostate cancer, and glioma [[21], [22], [23], [24], [25]]. Upregulation of GPC5 contrastively suppresses tumor migration, invasion, and proliferation in NSCLC [26,27]. Interestingly, a genetic variant of a high-risk allele linked to lower expression of GPC5 was defined in lung cancer patients who have never smoked [28]. Simultaneously, GPC5 expression was 50 % lower in adenocarcinoma than in matched healthy lung tissue [28]. Furthermore, loss of GPC5 induces tumor growth through Wnt/β-catenin signaling and correlates with poor outcomes in NSCLC [29]. These impaired expressions of GPC5 might be mediated by microRNAs (miRNAs), negative regulators of protein-coding genes. Some miRNAs, such as miR-297, miR-301b, miR-620, and miR-709, promote tumor malignancy by negative regulation of the GPC5-coding gene in cancer [23,24,30,31]. Thus, further investigations are essential to clarify the role of GPC5 as a tumor promoter or suppressor. Highly sensitive and specific antibodies are desired in multimodal experiments to analyze GPC5.

Previously, we have established numerous monoclonal antibodies (mAbs) against membrane proteins, including PD-L1 (clone L_1_Mab-13) [32], mouse CD39 (clone C_39_Mab-2) [33], EpCAM (clone EpMab-37) [34], and TROP2 (clone TrMab-6) [35] by using the Cell-Based Immunization and Screening (CBIS) method. The CBIS method includes the immunization of antigen-overexpressed cells and flow cytometry-based high-throughput screening. Using the CBIS method, we can efficiently obtain a wide variety of antibodies that recognize linear or structural epitopes and modifications of extracellular domains of membrane protein in a short period. In this study, we have successfully established a novel anti-human GPC5 mAb (clone G_5_Mab-1) that can be used for multiple applications using the CBIS method.

## 2 Materials and methods

### 2.1 Cell lines

Cell lines, including LN229, Chinese hamster ovary (CHO)–K1, and P3X63Ag8U.1 (P3U1) cells were obtained from the American Type Culture Collection (Manassas, VA, USA). The genes encoding human GPC5 (Catalog No.: IRAK082J20, Accession No.: NM_004466) were obtained from RIKEN BRC (Ibaraki, Japan). We thank Dr. Yoshihide Hayashizaki of RIKEN and Dr. Sumio Sugano of the University of Tokyo for providing the IRAK082J20 (cat. HGX033036) through the National BioResource Project of the MEXT, Japan. The expression plasmid of human GPC5 was subcloned into a pCAG-ble vector (FUJIFILM Wako Pure Chemical Corporation, Osaka, Japan). pCAG-hGPC5 vector was transfected into cell lines using the Neon transfection system (Thermo Fisher Scientific, Inc., Waltham, MA, USA). Subsequently, LN229 and CHO–K1, which stably overexpressed GPC5 (hereafter described as LN229/GPC5 and CHO/GPC5, respectively), were stained with an anti-GPC5 mAb (clone 297716; R&D Systems, Inc., Minneapolis, MN, USA) and sorted using the SH800 cell sorter (Sony corp., Tokyo, Japan), followed by cultivation in a medium containing 0.5 mg/mL of Zeocin (InvivoGen, San Diego, CA, USA).

The expression plasmid of GPC1 (pCMV6_GPC1, Catalog No.: SC321494, Accession No.: NM_002081) was purchased from OriGene Technologies, Inc. (Rockville, MD, USA). The complementary DNAs (cDNAs) of other Glypican members, including GPC2 (Catalog No.: IRAK049P06, Accession No.: NM_152742) and GPC4 (Catalog No.: IRAK015P17, Accession No.: NM_001448) were obtained from RIKEN RBC. GPC3v2 (Accession No.: NM_001164618) and GPC6 (Accession No.: NM_005708) cDNAs were synthesized by Eurofins Genomics KK (Tokyo, Japan). The cDNAs of GPC2, GPC3v2, and GPC4 were cloned into a pCAG-ble vector. A GPC6 cDNA was cloned into a pCAGzeo-ssnPA16 vector [36]. The plasmids were also transfected into CHO–K1 cells and stable transfectants were established by staining with an anti-GPC1 mAb (clone 1019718; R&D systems, Inc., Minneapolis, MN, USA), an anti-GPC2 (CT3) mAb (#90488; Cell Signaling Technology, Inc., Danvers, MA, USA), an anti-GPC3 mAb (clone ab95363; Abcam, Cambridge, UK), an anti-GPC4 mAb (clone A21050B; BioLegend, San Diego, CA, USA), and an anti-PA tag mAb (clone NZ-1 for GPC6) [36], and sorted using SH800, respectively. After sorting, cultivation in a medium containing 0.5 mg/mL of Zeocin (InvivoGen, San Diego, CA, USA) or 0.5 mg/mL of G418 (Nacalai Tesque, Inc.) was progressed. These GPCs-overexpressed CHO–K1 (e.g., CHO/GPC1) clones were finally established.

CHO–K1, P3U1, and GPCs-overexpressed CHO–K1 were also cultured in a Roswell Park Memorial Institute (RPMI)-1640 medium (Nacalai Tesque, Inc.) that was supplemented with 10 % heat-inactivated fetal bovine serum (FBS, Thermo Fisher Scientific Inc.), 100 units/mL penicillin, 100 μg/mL streptomycin, and 0.25 μg/mL amphotericin B (Nacalai Tesque, Inc.). LN229 and LN229/GPC5 were cultured in Dulbecco's Modified Eagle Medium (DMEM) (Nacalai Tesque, Inc.) that was supplemented with 10 % heat-inactivated FBS (Thermo Fisher Scientific Inc.), 100 units/mL penicillin, 100 μg/mL streptomycin, and 0.25 μg/mL amphotericin B (Nacalai Tesque, Inc.). Then, cells were cultured in a humidified CO_2_ incubator with 5% CO_2_ and 95% air at 37 °C.

### 2.2 Antibodies

An anti-Human/Mouse Glypican 5 Antibody (clone 297716, mouse IgG_2a_) was purchased from R&D Systems, Inc. An anti-isocitrate dehydrogenase 1 (IDH1) mAb (clone RcMab-1) was developed previously in our lab [37]. A secondary Alexa Fluor 488-conjugated anti-mouse, rat, and rabbit IgG was purchased from Cell Signaling Technology, Inc. (Danvers, MA, USA). Secondary horseradish peroxidase-conjugated anti-mouse IgG and anti-rat IgG were obtained from Agilent Technologies Inc. (Santa Clara, CA, USA) and Merck KGaA (Darmstadt, Germany), respectively. The sources of commercially available anti-GPC antibodies are listed above.

### 2.3 Hybridoma production

For developing anti-GPC5 mAbs, two 6-week-old female BALB/cAJcl mice, purchased from CLEA Japan (Tokyo, Japan), were immunized intraperitoneally with 1 × 10^8^ cells/mouse of LN229/GPC5. The LN229/GPC5 cells as immunogen were harvested after brief exposure to 1 mM ethylenediaminetetraacetic acid (EDTA; Nacalai Tesque, Inc.). Alhydrogel adjuvant 2% (InvivoGen, San Diego, CA, USA) was added as an adjuvant in the first immunization. Three additional injections of 1 × 10^8^ cells/mouse of LN229/GPC5 were administered intraperitoneally without an adjuvant addition every week. A final booster injection was performed with 1 × 10^8^ cells/mouse of LN229/GPC5 intraperitoneally two days before harvesting splenocytes from mice. We conducted cell-fusion of the harvested splenocytes from LN229/GPC5-immunized mice with P3U1 cells using polyethylene glycol 1500 (PEG1500; Roche Diagnostics, Indianapolis, IN, USA) under heated conditions.

Hybridomas were cultured in the RPMI-1640 medium supplemented as shown above, with additional supplements including hypoxanthine, aminopterin, and thymidine (HAT; Thermo Fisher Scientific, Inc.), 5% BriClone (NICB, Dublin, Ireland), and 5 μg/mL of Plasmocin (InvivoGen, San Diego, CA, USA) into the medium. The supernatants of hybridomas were screened by flow cytometric analysis using CHO/GPC5 and parental CHO–K1 cells. The hybridoma supernatant, containing G_5_Mab-1 in serum free-medium, was filtrated and purified using Ab-Capcher Extra (ProteNova, Kagawa, Japan).

### 2.4 Flow cytometry

Cells were harvested using 1 mM EDTA. Subsequently, cells were washed with 0.1% bovine serum albumin in phosphate-buffered saline (PBS) and treated with primary mAbs for 30 min at 4 °C. Afterward, cells were treated with Alexa Fluor 488-conjugated anti-mouse IgG (1:1000) following the collection of fluorescence data using the SA3800 Cell Analyzer (Sony Corp.). Expression of GPCs in GPCs-overexpressed CHO–K1 cells was confirmed with specific antibodies, 0.5 μg/mL of an anti-GPC1 mAb (1019718) for CHO/GPC1, 0.5 μg/mL of an anti-GPC2 mAb (#90488) for CHO/GPC2, 0.466 μg/mL of an anti-GPC3 mAb (ab95363) for CHO/GPC3, 1.2 μg/mL of an anti-GPC4 mAb (A21050B) for CHO/GPC4, 0.5 μg/mL of an anti-GPC5 mAb (297716) for CHO/GPC5, 1 μg/mL of an anti-PA tag mAb (NZ-1) for CHO/GPC6, respectively.

### 2.5 Determination of the binding affinity by flow cytometry

CHO/GPC5 cells were suspended in 100 μL serially diluted G_5_Mab-1 (30 μg/mL to 0.002 μg/mL) and 297716 (an anti-human/mouse GPC5 mAb, 30 μg/mL to 0.002 μg/mL) after which Alexa Fluor 488-conjugated anti-mouse IgG (1:200) was treated. Fluorescence data were subsequently collected using the SA3800 Cell Analyzer, following the calculation of the dissociation constant (*K*_D_) by fitting the binding isotherms into the built-in one-site binding model in GraphPad PRISM 6 (GraphPad Software, Inc., La Jolla, CA, USA).

### 2.6 Western blot analysis

Cell lysates were boiled in sodium dodecyl sulfate (SDS) sample buffer (Nacalai Tesque, Inc.). Proteins (10 μg/lane) were electrophoresed on 5%–20% polyacrylamide gels (Wako) and transferred onto polyvinylidene difluoride (PVDF) membranes (Merck KGaA). After blocking with 4 % non-fat milk (Nacalai Tesque, Inc.), PVDF membranes were incubated with 5 μg/mL of G_5_Mab-1, 5 μg/mL of 297716, 1 μg/mL of an anti-IDH1 mAb (RcMab-1), followed by incubation with horseradish peroxidase-conjugated anti-mouse IgG (1:2000; Agilent Technologies Inc.) or anti-rat IgG (1:10000; Merck KGaA). Chemiluminescence signals were developed using Pierce™ ECL Plus (Thermo Fisher Scientific, Inc.) and ImmunoStar LD (Wako). The signals were imaged with a Sayaca-Imager (DRC Co. Ltd., Tokyo, Japan).

### 2.7 Immunohistochemical analysis

The CHO/GPC5 and CHO–K1 cell blocks were prepared using iPGell (Genostaff Co., Ltd., Tokyo, Japan). The paraffin-embedded cell sections were autoclaved in a citrate buffer (pH 6.0; Nichirei Biosciences, Inc., Tokyo, Japan). After blocking using the SuperBlock T20 Blocking Buffer (Thermo Fisher Scientific Inc.), the sections were incubated with 1 μg/mL of G_5_Mab-1 and 1 μg/mL of 297716 and then treated with the Envision + Kit (Agilent Technologies Inc.). Color was developed using 3,3′-diaminobenzidine tetrahydrochloride (Agilent Technologies Inc.), and counterstaining was performed using hematoxylin (Merck KGaA, Darmstadt, Germany).

## 3 Results

### 3.1 Development of anti-GPC5 mAbs using the CBIS method

To establish anti-GPC5 mAbs, we employed the CBIS method using GPC5-overexpressed cells. Anti-GPC5 mAbs-producing hybridomas were screened by using flow cytometry (Fig. 1). Two female BALB/cAJcl mice were intraperitoneally immunized with LN229/GPC5 (1 × 10^8^ cells/time/mouse) every week, 5 times. Subsequently, mouse splenocytes and P3U1 cells were fused by PEG1500. Hybridomas were seeded into 96-well plates, after which the flow cytometric screening was conducted to select CHO/GPC5-reactive and parental CHO–K1-nonreactive supernatants of hybridomas. We obtained some highly CHO/GPC5-reactive supernatants of hybridomas. We finally established the highly sensitive clone G_5_Mab-1 (mouse IgG_1_, kappa) by limiting dilution and additional analysis.

**Fig. 1:**
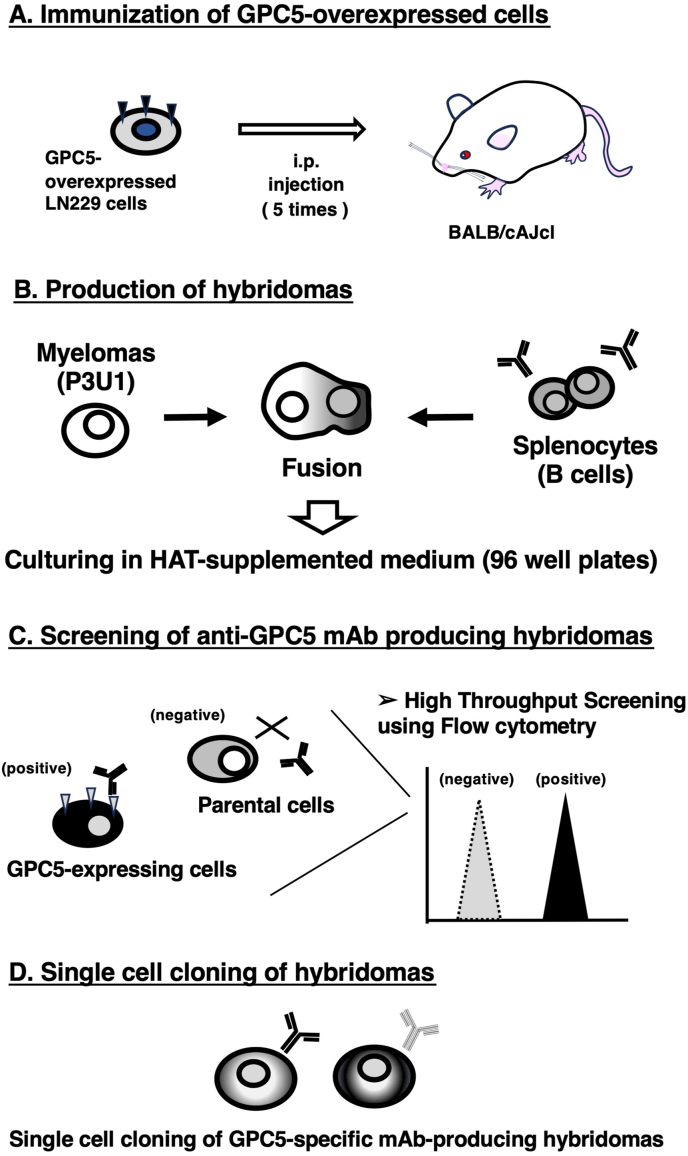
A schematic depiction of the CBIS method for developing anti-GPC5 mAbs. The simplified procedure flow of mAb development using the CBIS method. (A) LN229/GPC5 cells were intraperitoneally immunized into two female BALB/cAJcl mice. (B) The spleen cells from antigen-immunized mice were fused with P3U1 myeloma cells by PEG1500. (C) The culture supernatants of hybridoma were screened by flow cytometry using CHO–K1 and CHO/GPC5 to select GPC5-specific mAb-producing hybridomas. (D) Single hybridoma clones were obtained by limiting dilution, followed by additional screening. Finally, G_5_Mab-1 (mouse IgG_1_, kappa) was successfully established.

### 3.2 Evaluation of antibody reactivity and specificity using flow cytometry

**Fig. 2:**
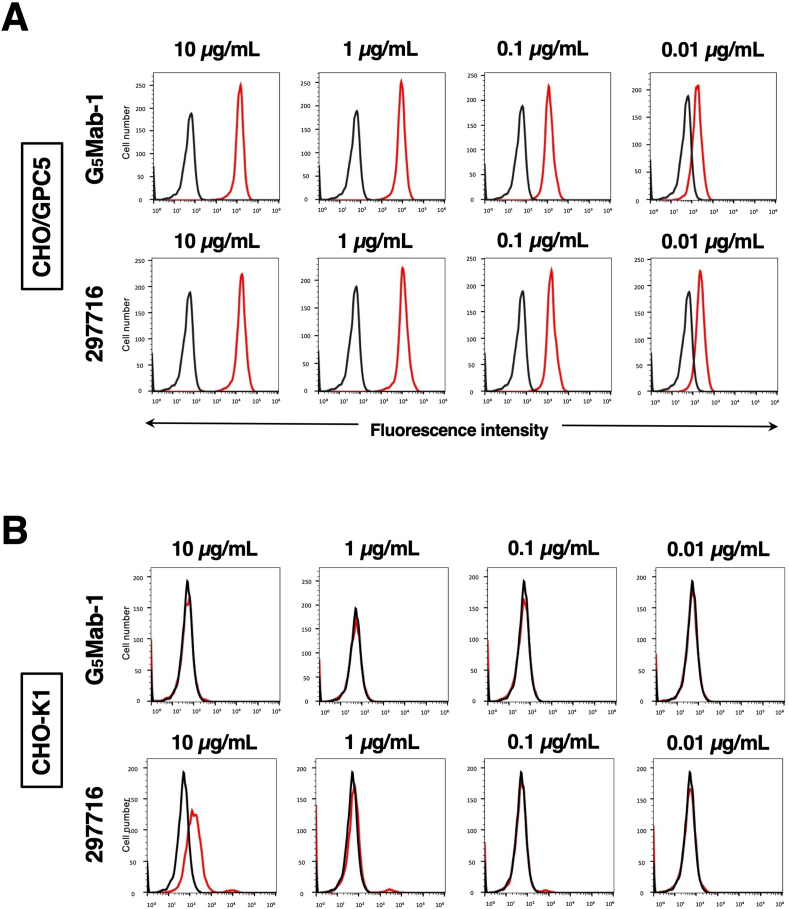
Flow cytometric analysis of anti-GPC5 mAbs. CHO/GPC5 (A), CHO–K1 (B) cells were treated with 0.01–10 μg/mL of G_5_Mab-1 or 297716 (red line), followed by treatment with Alexa Fluor 488-conjugated anti-mouse IgG. Fluorescence data were collected using the SA3800 Cell Analyzer. Black line, control (no primary antibody treatment).

Flow cytometric analysis was conducted using G_5_Mab-1 and a commercially available anti-human/mouse GPC5 mAb (clone 297716) against CHO–K1 and CHO/GPC5 cells. Results indicated that G_5_Mab-1 and 297716 recognized CHO/GPC5 dose-dependently (Fig. 2A). Reactivity is almost identical between G_5_Mab-1 and 297716 to CHO/GPC5 (Fig. 2A). G_5_Mab-1 did not react with parental CHO–K1 cells even at a concentration of 10 μg/mL (Fig. 2B). However, 297716 showed the reaction with CHO–K1 cells at a concentration of 10 μg/mL (Fig. 2B). Thus, G_5_Mab-1 can detect GPC5, more specifically in flow cytometry.

**Fig. 3:**
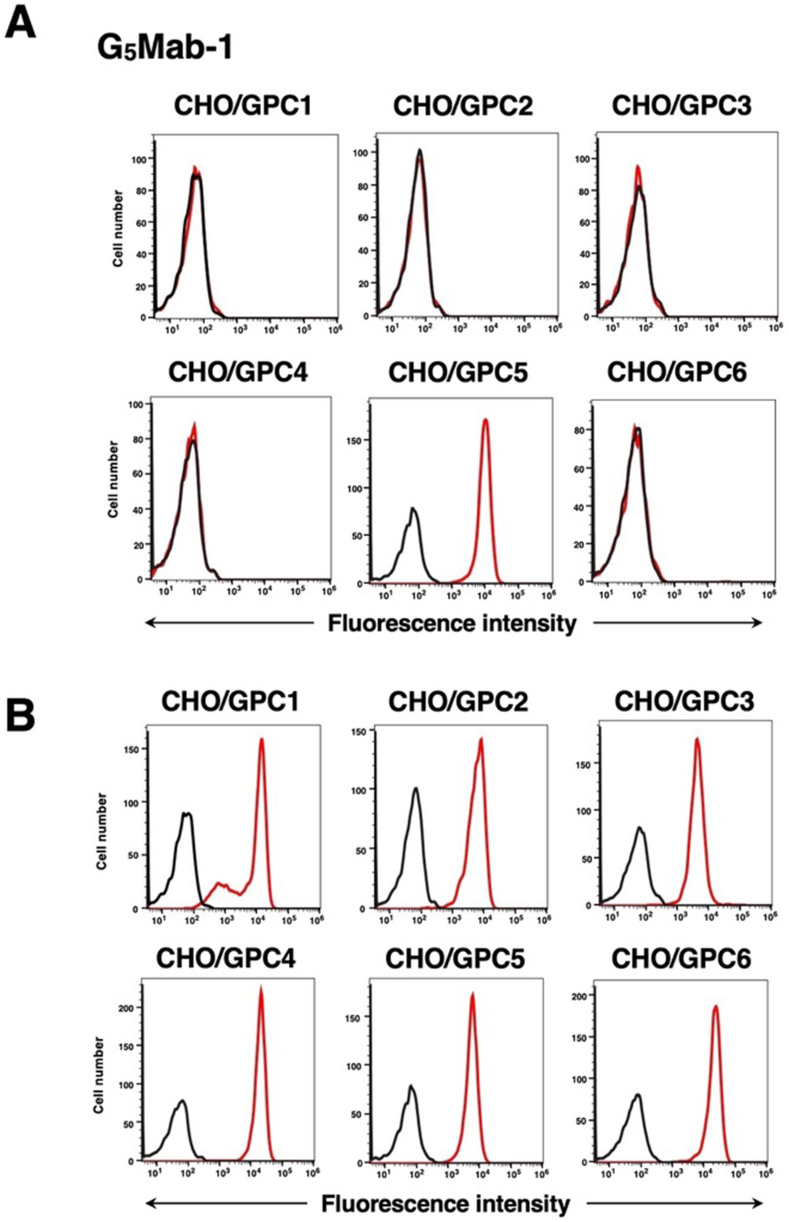
Flow cytometry of G_5_Mab-1 in GPCs-expressed CHO–K1 cells. CHO–K1 cells, which overexpressed each of the six GPCs, were treated with G_5_Mab-1 (10 μg/mL) (A) and specific antibodies for each GPCs as described in section 2.4 (B) (red line). The black line shows the cells with control-blocking buffer treatment instead of primary antibody. After incubation with primary antibody or control blocking buffer, anti-mouse, rat, or rabbit IgG conjugated with Alexa Fluor 488 was treated. Fluorescence data were collected using the SA3800 Cell Analyzer.

### 3.3 Specificity of G_5_Mab-1 to Glypican-overexpressed CHO–K1 cells

We have also established five other cell lines of GPCs-overexpressed CHO–K1 cells, such as CHO/GPC1, CHO/GPC2, CHO/GPC3, CHO/GPC4, and CHO/GPC6. Using the six cell lines, the specificity of G_5_Mab-1 was analyzed. As shown in Figs. 3A, 10 μg/mL of G_5_Mab-1 potently recognized CHO/GPC5, but not others. The expression of GPCs in all cell lines was confirmed by specific antibodies (Fig. 3B).

### 3.4 Calculation of the apparent binding affinity of anti-GPC5 mAbs using flow cytometry

**Fig. 4:**
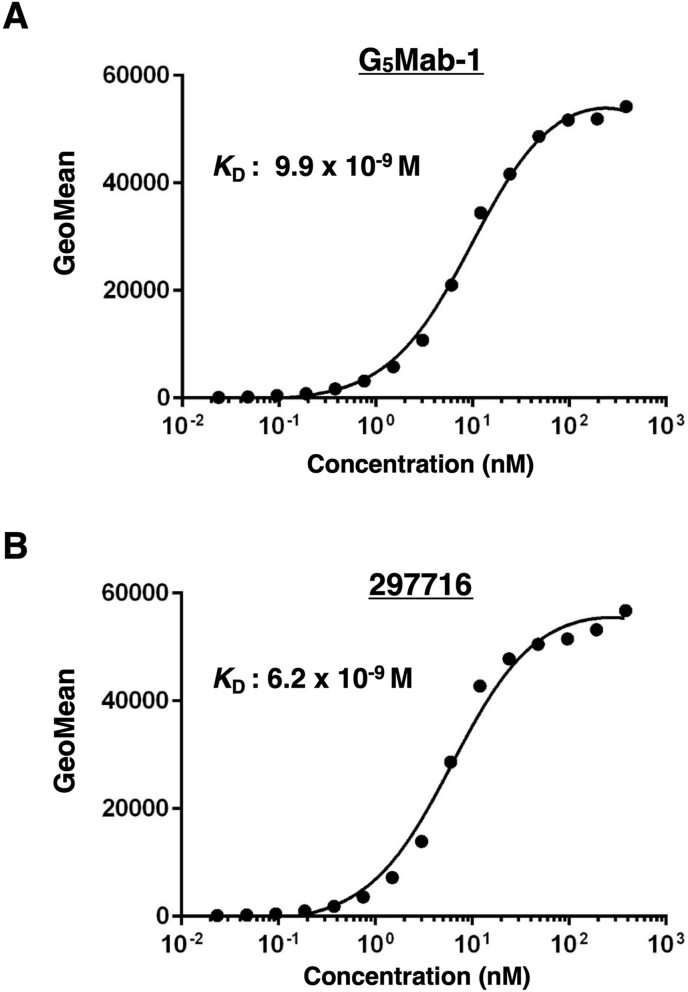
Determination of the binding affinity of G_5_Mab-1. CHO/GPC5 cells were suspended in 100 μL of serially diluted 30 μg/mL to 0.002 μg/mL of G_5_Mab-1 (A) or 30 μg/mL to 0.002 μg/mL of 297716 (B). Then, cells were treated with Alexa Fluor 488-conjugated anti-mouse IgG. Subsequently, the geometric mean values from fluorescence data were collected using the SA3800 Cell Analyzer, following the calculation of the *K*_D_ by GraphPad PRISM 6 software.

The binding affinity of G_5_Mab-1 and 297716 was assessed with CHO/GPC5 using flow cytometry. The results indicated that the *K*_D_ value of G_5_Mab-1 was 9.9 × 10^−9^ M (Fig. 4A). The *K*_D_ value of 297716 was 6.2 × 10^−9^ M (Fig. 4B). There was no noticeable difference in binding affinity for CHO/GPC5 between G_5_Mab-1 and 297716. These results demonstrate that G_5_Mab-1 can recognize GPC5 with moderate affinity to cell surface GPC5.

### 3.5 Western blot analyses using anti-GPC5 mAbs

**Fig. 5:**
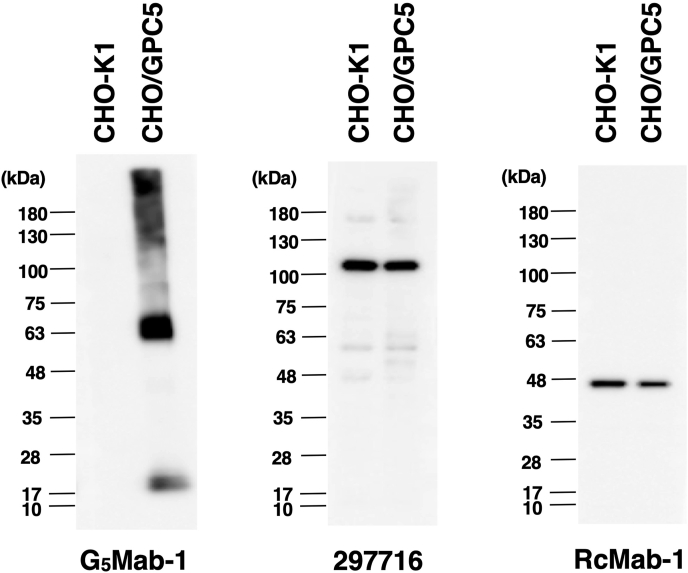
The detection of GPC5 by Western blot analysis. Cell lysates of CHO–K1 and CHO/GPC5 (10 μg/lane) were electrophoresed and transferred onto PVDF membranes. The membranes were incubated with 1 μg/mL of G_5_Mab-1, 1 μg/mL of 297716, and 1 μg/mL of RcMab-1 and subsequently with horseradish peroxidase-conjugated anti-mouse or anti-rat immunoglobulins.

We investigated whether G_5_Mab-1 can be used for Western blot analysis by analyzing CHO–K1 and CHO/GPC5 cell lysates. The estimated molecular weight of the GPC5 protein is approximately 60 kDa. As shown in Fig. 5, G_5_Mab-1 could detect GPC5 as the major band around 63 kDa in CHO/GPC5 cell lysates, while no band was detected in parental CHO–K1 cells. Another anti-GPC5 mAb (clone 297716) could not detect GPC5 as the band around 63 kDa in CHO/GPC5 cell lysates. An anti-IDH1 mAb (clone RcMab-1) was used for internal control. These results indicate that G_5_Mab-1 can detect GPC5 in GPC5-overexpressing cells in Western blot analyses.

### 3.6 Immunohistochemistry using anti-GPC5 mAbs

**Fig. 6:**
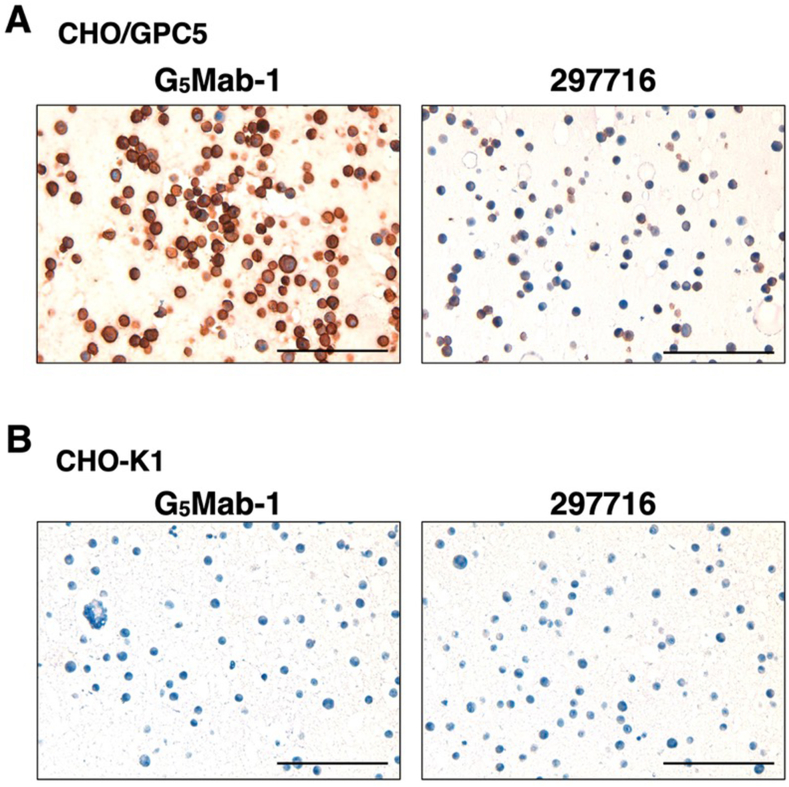
Immunohistochemical staining of paraffin-embedded section of CHO/GPC5 and CHO–K1. The sections of CHO/GPC5 (A) and CHO–K1 (B) cells were treated with 1 μg/mL of G_5_Mab-1 or 1 μg/mL of 297716, followed by that with the Envision + Kit. Color was developed using DAB, and counterstaining was performed using hematoxylin (Merck KGaA, Darmstadt, Germany). Scale bar = 100 μm.

To investigate whether G_5_Mab-1 can be used for immunohistochemistry (IHC), paraffin-embedded CHO–K1 and CHO/GPC5 sections were stained with G_5_Mab-1. Apparent membranous staining by G_5_Mab-1 was observed in CHO/GPC5 (Fig. 6A). The 297716, another anti-GPC5 mAb, partially and weakly stained CHO/GPC5 sections (Fig. 6A). Both G_5_Mab-1 and 297716 did not react with the CHO–K1 section. (Fig. 6B). These results indicate that G_5_Mab-1 applies to IHC for detecting GPC5-positive cells in paraffin-embedded cell samples.

### 3.7 Determination of complementarity-determining regions of G_5_Mab-1

We cloned the cDNA of G_5_Mab-1 variable regions and showed amino acid sequences of complementarity-determining regions (Supplementary Fig. 1).

## 4 Discussion

We successfully developed a novel anti-human GPC5 mAb clone G_5_Mab-1 by the CBIS method. G_5_Mab-1 showed almost the same reactivity in flow cytometric analysis as 297716, a commercially available anti-GPC5 mAb (Fig. 2, Fig. 4). G_5_Mab-1 specifically recognized GPC5 but not other members of the GPC family (Fig. 3). Furthermore, in Western blot and immunohistochemistry, G_5_Mab-1 clearly detected GPC5 under denaturing conditions (Fig. 5, Fig. 6). Contrary to the supplier data of 297716, we could not detect GPC5 in the Western blot (Fig. 5). In immunohistochemical analyses, the sensitivity of the 297716 was much lower than that of the G_5_Mab-1 (Fig. 6). Therefore, G_5_Mab-1 is a versatile mAb and will contribute to the biological analysis of GPC5 and its diagnosis. Some reports have described that GPC5 overexpression promotes tumor malignancy [6,16,38]. Therefore, GPC5 may be a therapeutic target for cancer. To evaluate *in vivo* antitumor activity of G_5_Mab-1, we will convert G_5_Mab-1 (mouse IgG_1_) to IgG_2a_ version to confer antibody-dependent cellular cytotoxicity activity, and evaluate the antitumor efficacy in xenograft models, as described previously [[39], [40], [41]].

GPC3 is currently considered the most promising cancer antigen in the GPC family. Codrituzumab (GC33), an anti-GPC3 mAb, has been treated in advanced hepatocellular carcinoma (HCC) patients in a phase I clinical study. Codrituzumab treatment was effective in some patients with high GPC3 expression, but no benefit was obtained in the subsequent phase II trials [[42], [43], [44]]. A chimeric antigen receptor-T (CAR-T) targeting GPC3 has also been developed for cancer treatment [45]. No treatments targeting GPC5 have been reported yet. Within the GPC family, the amino acid sequence homology between GPC3 and GPC5 is 43%, and the N-terminus shows a relatively high homology of 54% [46]. The C-terminal Ser-Gly repeating glycosylation sites between GPC3 and GPC5 are also similarly positioned [14]. Like GPC5, GPC3 regulates many cancer-progressive cascades, including Wnt, hedgehog, and FGF [[47], [48], [49], [50]]. Although further verification is required, these findings suggest that GPC5 may become one of the critical regulators of cancer. G_5_Mab-1 can be a valuable tool for versatile analysis of GPC5 in cancer research.

In addition to heparan sulfate attachment, GPC1 and GPC3 have sites for *N*-glycosylation in the extracellular domain [51,52]. Glycosylation of proteins often regulates cell functions through protein folding, stability, and signaling. Aberrant glycosylation is involved in the progression of diseases [53]. Considering the amino acid homology among the GPC family, glycosylation might also affect the function of GPC5. Previously, we have developed a cancer-specific mAb targeting podoplanin (PDPN) clone LpMab-2 by immunizing LN229 glioblastoma cells-derived PDPN [54]. The epitope of LpMab-2 includes *O*-glycosylation of PDPN expressed in cancer cells. Using a similar method to produce mAb against GPC5 may clarify the relationship between GPC5 and glycosylation in cancers. Furthermore, we have successfully established a cancer-specific anti-human epidermal growth factor receptor 2 (HER2) mAb, clone H_2_Mab-214, by immunizing LN229-producing HER2 ectodomain [55]. The H_2_Mab-214 recognizes disrupted structure in HER2 domain IV, a cancer-specific epitope, rather than glycosylation. In future studies, we intend to identify whether cancer-specific GPC5 structures and modifications exist by further developing anti-GPC5 mAbs using the same strategy.

## CRediT authorship contribution statement

**Yu Kaneko:** Project administration, Conceptualization, Writing – review & editing, Funding acquisition. **Tomohiro Tanaka:** Writing – original draft, Funding acquisition, Investigation. **Shiori Fujisawa:** Investigation. **Guanjie Li:** Investigation. **Hiroyuki Satofuka:** Funding acquisition, Writing – review & editing. **Mika K. Kaneko:** Conceptualization. **Hiroyuki Suzuki:** Investigation, Funding acquisition. **Yukinari Kato:** Conceptualization, Funding acquisition, Project administration, Writing – review & editing.

## Author disclosure statement

The authors have no conflict of interest.

## Funding information

This research was supported in part by 10.13039/100009619Japan Agency for Medical Research and Development (AMED) under Grant Numbers: JP24am0521010 (to Y.Kato), JP24ama121008 (to Y.Kato), JP24ama221339 (to Y.Kato), JP24bm1123027 (to Y.Kato), and JP24ck0106730 (to Y.Kato), and by the 10.13039/501100001691Japan Society for the Promotion of Science (JSPS) Grants-in-Aid for Scientific Research (KAKENHI) grant nos. 22K06995 (to H.Suzuki), 24K18268 (to T.T.), 24K11652 (to H.Satofuka), and 25K10553 (to Y.Kato).

## Declaration of competing interest

The authors declare that they have no known competing financial interests or personal relationships that could have appeared to influence the work reported in this paper.

## Figures and Tables

**figs1:**
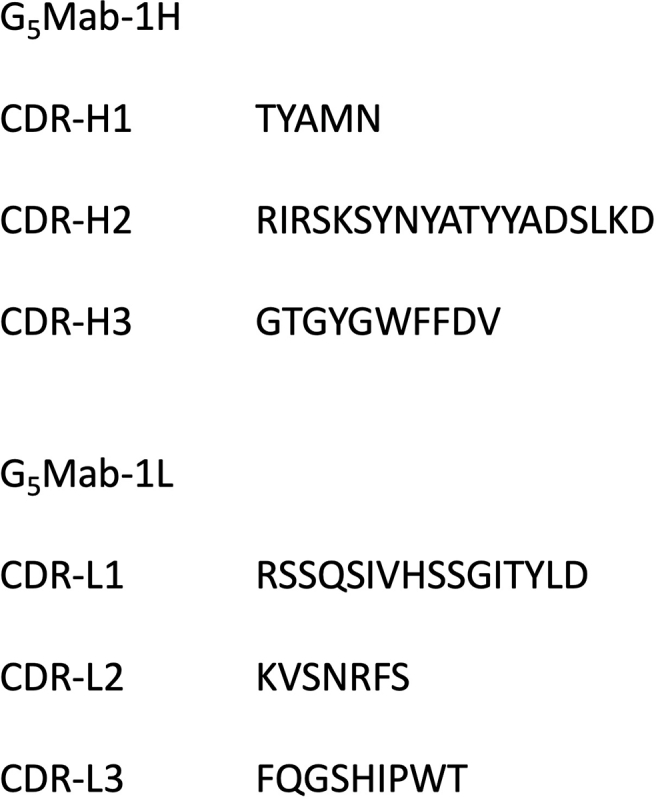
Supplementary Fig. 1 Amino acid sequence of complementarity-determining regions of G_5_Mab-1. The cDNA of G_5_Mab-1 variable regions was cloned, and the complementarity-determining region (CDR) of heavy chain (G_5_Mab-1H) and light chain (G_5_Mab-1L) were determined.

**figs2:**